# Plant Response to Cold Stress: Cold Stress Changes Antioxidant Metabolism in Heading Type Kimchi Cabbage (*Brassica rapa* L. ssp. Pekinensis)

**DOI:** 10.3390/antiox11040700

**Published:** 2022-04-01

**Authors:** Seung Hee Eom, Min-A Ahn, Eunhui Kim, Hee Ju Lee, Jin Hyoung Lee, Seung Hwan Wi, Sung Kyeom Kim, Heung Bin Lim, Tae Kyung Hyun

**Affiliations:** 1 Department of Industrial Plant Science and Technology, College of Agricultural, Life and Environmental Sciences, Chungbuk National University, Cheongju 28644, Korea; eom0214@naver.com (S.H.E.); koala0523@chungbuk.ac.kr (M.-A.A.); eh981102@gmail.com (E.K.); 2 Vegetable Research Division, National Institute of Horticultural & Herbal Science, Wanju 55365, Korea; perpetuaa@korea.kr (H.J.L.); leejh0820@korea.kr (J.H.L.); kgad@korea.kr (S.H.W.); 3 Department of Horticultural Science, College of Agriculture and Life Science, Kyungpook National University, Daegu 41566, Korea; skkim76@knu.ac.kr

**Keywords:** antioxidant, kimchi cabbage, cold stress, phenylpropanoid pathway

## Abstract

Cold stress is known as the important yield-limiting factor of heading type Kimchi cabbage (HtKc, *Brassica rapa* L. ssp. pekinensis), which is an economically important crop worldwide. However, the biochemical and molecular responses to cold stress in HtKc are largely unknown. In this study, we conducted transcriptome analyses on HtKc grown under normal versus cold conditions to investigate the molecular mechanism underlying HtKc responses to cold stress. A total of 2131 genes (936 up-regulated and 1195 down-regulated) were identified as differentially expressed genes and were significantly annotated in the category of “response to stimulus”. In addition, cold stress caused the accumulation of polyphenolic compounds, including p-coumaric, ferulic, and sinapic acids, in HtKc by inducing the phenylpropanoid pathway. The results of the chemical-based antioxidant assay indicated that the cold-induced polyphenolic compounds improved the free-radical scavenging activity and antioxidant capacity, suggesting that the phenylpropanoid pathway induced by cold stress contributes to resistance to cold-induced reactive oxygen species in HtKc. Taken together, our results will serve as an important base to improve the cold tolerance in plants via enhancing the antioxidant machinery.

## 1. Introduction

According to Intergovernmental Panel on Climate Change (IPCC; www.ipcc.ch, accessed on 30 March 2022), the instability of global temperatures is accompanied by an increase in the frequency and intensity of heatwaves and cold spells, which can have negative effects on crop yields. Cold stress, classified as chilling stress (<20 °C) and freezing stress (<0 °C), has become a major environmental factor that influences life cycles and the geographical distributions of plants, as well as crop yields, by affecting plant growth, development, and flowering time [1]. The physical phase transition of the cell membrane from the liquid crystalline phase to the gel-like solid phase is known as the primary event resulting from chilling stress and causes insufficient energy, ion leakage, and accumulation of reactive oxygen species (ROS) [2]. Excessive ROS levels lead to membrane disruption and eventually cell death. To protect plants from chilling injury, various chemicals, such as polyamines, γ-aminobutyric acid (GABA), methyl jasmonate (MeJA), and ethylene, have been applied [3]. Among them, GABA increases the activities of both enzymatic and non-enzymatic antioxidants in the carambola fruit and thereby attenuates chilling injury [3]. Similarly, MeJA improves the chilling tolerance of the eggplant fruit by enhancing the activities and related gene expression of antioxidant enzymes, including catalase (CAT) and peroxidase (POD) [4]. In addition, plants overexpressing superoxide dismutase (SOD) exhibit increased tolerance against chilling stress in comparison with that of wild-type plants [5]. These indicate that the antioxidant machinery plays an essential role in chilling tolerance, suggesting that particular attention should be paid to the antioxidant machinery for successful yield protection against cold stress.

In Korea, heading type Kimchi cabbage (HtKc, *Brassica rapa* L. ssp. pekinensis) is mostly used as the principal ingredient of kimchi, and its production value amounted to over six billion USD in 2015 [6]. Although HtKc is known as a cool-season crop (optimal growth temperature, 18–20 °C; the lowest growth temperature, 4–5 °C) [7], cold stress (< 10 °C) affects its growth and development, as well as production yield [6,8]. Since the reference genome of the Chinese cabbage was successfully completed in 2011, *Brassica rapa* cold-responsive genes (*BrCRGs*), including heat stress transcription factors, DEAD-box RNA helicases, and stress-inducible proteins, have been identified via the full-length cDNA microarray and expressed sequence tags analyses [9,10]. Using winter and summer seasonal cultivars of the Chinese cabbage, *BrCRG1*-*7* have been identified as candidate cold-tolerance genes [9]. In addition, based on genome-wide analysis, multiple genes, including cold-shock-domain proteins, cold-shock proteins, the bHLH transcription factor *ICE1*, the *ZF-HD* gene family, and protein disulfide isomerases, have been identified as cold-response genes in the Chinese cabbage [11,12,13,14,15], providing a basis for further understanding of cold response in Chinese cabbage. However, cold-induced transcriptomic changes, including genes involved in the antioxidant machinery, remain largely unknown in HtKc.

In this study, we determined the expression of key genes and the activities of the antioxidant machinery under cold stress through a transcriptomic approach. Gene expression profiling was carried out, and functional annotations of differentially expressed genes (DEGs) provided an overview of the cold stress response in HtKc. In addition, the analysis of the antioxidant machinery revealed the involvement of the phenylpropanoid pathway in controlling the cellular redox homeostasis under cold stress. Taken together, this report provides an overview of molecular responses triggered by cold stress in HtKc and will serve as a publicly available resource for future studies to improve environmental-stress tolerance via manipulation of the antioxidant machinery.

## 2. Materials and Methods

### 2.1. Plants and Stress Treatment

Seeds of HtKc (cultivar Chunkwang, Sakata Korea Seed Co., Ltd., Seoul, Korea) were germinated and grown in a glasshouse (20 °C). The seedlings grew in a uniform status for 50 d and were then transferred into extreme-weather growth chambers (EGC, Chagrin Falls, OH, USA) for cold treatment. Half of the plants were grown at 20 °C as control plants, and the remaining were subjected to cold stress (10 °C) for 1 day (C1d) or 3 days (C3d). The experiment was conducted with five biological replicates.

### 2.2. Physiological Response of HtCc to Cold Stress

The effects of cold stress on the photosynthetic rate and stomatal conductance were analyzed using the portable LI-COR gas-exchange system (LI-COR, Inc., Lincoln, NE, USA), as described by Lee et al. [6].

The proline content was quantitated using a colorimetric assay, as described by Abrahám et al. [16]. The proline amount was determined using a calibration curve prepared using various concentrations of a proline standard and expressed as ng/mg of fresh weight (F.W.).

The expression pattern of *BrCRGs* was analyzed using qRT-PCR as described below (Section 2.5).

### 2.3. Analysis of Activities of Antioxidant Enzymes

For analysis of SOD, CAT, and POD activities, total protein was extracted using 20 mM potassium phosphate buffer (pH 6.5) and quantitated according to the Bradford method [17]. SOD, CAT, and POD activities were analyzed using a SOD Assay Kit-WST (Sigma-Aldrich, St. Louis, MO, USA), Amplex Red Catalase Assay Kit (Molecular Probes, Eugene, OR, USA), and Amplex^™^ Red Hydrogen Peroxide/Peroxidase Assay Kit (Invitrogen, Carlsbad, CA, USA), respectively.

### 2.4. Transcriptome Analysis

Equal amounts of total RNA from the independent replicates were pooled, and the cDNA library was generated using mRNA fragments, as described by Choi et al. [18]. Paired-end sequencing was performed using the Illumina platform. Clean reads were obtained, as described by Eom et al. [19] and then mapped to the *B*. *rapa* (subsp. pekinensis, inbred line Chiifu-401-42) reference sequence (BrapaV3.0), as described by Eom et al. [19]. Transcript levels were expressed as fragments per kilobase of transcript per million reads mapped using HTSeq-count and DESeq2, as described by Hong et al. [20]. DEGs were determined based on a *p*-value cutoff of 0.01 and |log2 (fold change)| ≥ 1. The expression levels of the DEGs were shown as Z-scores of FPKM values [20].

Blast2GO (https://www.blast2go.com/, accessed on 21 August 2021) was used for gene ontology (GO) enrichment of the DEGs. In addition, metabolism overview maps were drawn using MapMan (http://mapman.gabipd.org/, accessed on 26 September 2021) [19].

### 2.5. qRT-PCR Analysis

To verify the expression patterns observed in the transcriptomic data, genes involved in photosystem II (PSII) and I (PSI) were selected for qRT-PCR analysis. The transcription levels of genes were normalized to the internal reference gene actin [19]. The primer sequences used for qRT-PCR are listed in Table S1.

### 2.6. Analysis of the Non-Enzymatic Antioxidant Capacities of HtCc Extracts

Freeze-dried samples were soaked in MeOH for 24 h at room temperature. After filtration, the MeOH extracts were evaporated using a rotary vacuum evaporator. The free-radical scavenging activity of each extract was determined using the 1,1-diphenyl-2-picrylhydrazyl (DPPH) assay, and the oxygen-radical antioxidant capacity (ORAC) assay was performed, both as described by Kim et al. [21]. DPPH values were expressed as concentrations required to reduce half of the DPPH free radicals (IC_50_), and ORAC values were expressed as µM of Trolox equivalents (µM TE).

### 2.7. HPLC Analysis

The contents of p-coumaric, ferulic, and sinapic acids were determined using HPLC equipped with a diode array detector (Agilent Technologies, Waldbronn, Germany) and Poroshell 120 EC-c18 column (4.6 × 150 mm, 4 µm). The mobile phases consisted of 0.1% formic acid in distilled water (mobile phase A) and acetonitrile containing 0.1% formic acid (mobile phase B). The contents of p-coumaric, ferulic, and sinapic acids in each extract were determined by comparing the retention times and UV spectral data of the samples with those of the standards.

### 2.8. Statistical Analysis

Significant differences between the groups were determined using one-way ANOVA followed by Duncan’s multiple range test. *p*-values < 0.05 were considered to indicate statistical significance.

## 3. Results and Discussion

### 3.1. Physiological Response of HtKc to Cold Stress

**Figure 1:**
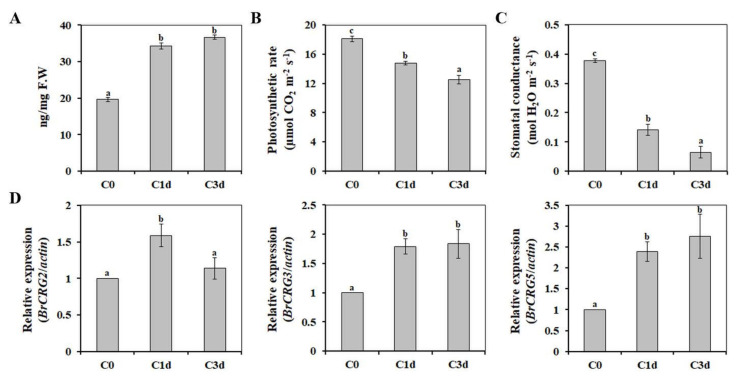
Physiological response to cold stress in heading type Kimchi cabbage. The changes in levels of proline (**A**), photosynthetic rate (**B**), stomatal conductance (**C**), and selected gene expression (**D**) after cold treatment were determined. The data are presented as mean ± SE. Values with different letters are significantly different, according to Duncan’s multiple range test. C0, plants grown at 20 °C (control plants); C1d and C3d, plants subjected to cold stress (10 °C) for 1 day and 3 days, respectively.

Cold response and tolerance of plants can vary substantially, depending on the treatment, developmental stage, and cold acclimation [22]. In higher plants, proline has been proposed to serve as the main osmolyte to prevent water loss [23], and its accumulation is known as a common physiological response of plants to cold stress [24]. To evaluate the efficacy of the treatment, we evaluated the cold-induced physiological changes, including proline content. As shown in Figure 1A, cold stress significantly induced the accumulation of proline in HtKc, indicating that temperature stress at 10 °C induced cold-response in HtKc.

Photosynthesis is highly sensitive to cold stress, which inhibits photochemical activities and impairs the balance between the absorption and utilization of light energy [25]. As in other plants, cold stress decreased the photosynthetic rate (Figure 1B) and stomatal conductance in HtKc (Figure 1C). In addition, we determined the transcription levels of the cold-responsive genes *BrCRG2*, *3*, and *5*. When HtKc plants were treated with cold stress, we observed up-regulation of all three genes (Figure 1D). These results indicated that the cold treatment was effective. The leaves of the HtKc plants grown under control (C0) versus cold-stress (C1d and C3d) conditions were harvested to analyze the molecular basis of the cold-stress response in HtKc.

### 3.2. Analysis of the Transcriptomic Changes in Response to Cold Stress

**Figure 2:**
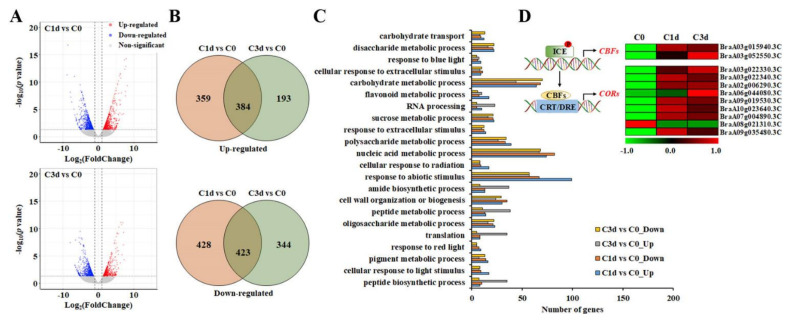
Cold-induced differentially expressed genes (DEGs) in heading type Kimchi cabbage. Volcano plot (**A**) and Venn diagram (**B**) analyses of the DEGs (C1d vs. C0, and C3d vs. C0). Gene ontology enrichment analysis of the DEGs (**C**), based on 2131 DEGs. (**D**) Expression heatmap showing the Z-scores of the DEGs involved in the ICE-CBF-COR cascade. C0, plants grown at 20 °C (control plants); C1d and C3d, plants subjected to cold stress (10 °C) for 1 day and 3 days, respectively. The color scale represents the raw Z-score ranging from red to green. Red and green colors indicate ascending and descending Z-scores, respectively.

Transcriptome sequencing has proven to be a highly efficient strategy to unravel the global expression network in adversity stress in many species, such as *B*. *rapa* [19] and *B*. *rapus* [26,27]. To investigate an overview of the molecular mechanisms associated with the cold-stress response in HtKc, we generated transcriptome libraries using total RNA obtained from the leaves of control and cold-stressed HtKc, and transcriptome libraries were deposited in the National Agricultural Biotechnology Information Center (http://nabic.rda.go.kr, accessed on 28 February 2022, Table S2). After removing the low-quality reads, 33–35 million clear reads (3.16 to 3.34 Gb) were obtained from three libraries, and >90% of the clean reads could be mapped on the reference genome (Table S2). To identify the cold-induced DEGs, we constructed two comparison groups (C1d vs. C0 and C3d vs. C0), and the DEGs were determined based on the cutoff used (*p*-value ≤ 0.01 and |log2 (fold change)| ≥ 1), as indicated on a Volcano plot (Figure 2A). A total of 1594 DEGs, including 743 up-regulated and 851 down-regulated genes, were detected between C0 and C1d libraries (Figure 2B). Comparative analysis of C0 and C3d libraries exhibited that 577 genes were up-regulated, and 767 genes were down-regulated in C3d. Among them, 384 up-regulated and 423 down-regulated genes were commonly detected in cold-stressed HtKc. To understand the biological function of DEGs, GO enrichment analysis was carried out. Various functional groups were identified, and a large proportion of the DEGs were found involved in the “carbohydrate metabolic process”, “response to abiotic stimulus”, and “nucleic acid metabolic process” (Figure 2C), indicating that the cold stress affected various metabolic processes. Similar to our findings, the GO term “response to abiotic stimulus” has been highlighted by various transcriptomic studies related to cold stress in other plants [28,29,30]. To date, a significant number of cold-response genes have been identified in numerous crops. Among them, the inducer of CBF expression (ICE)-C-repeat binding factor (CBF)-cold-responsive (COR) pathway is one of the most commonly reported pathways [31]. CBF genes, activated by ICE, up-regulate COR genes to enhance plant cold tolerance [31]. In HtKc, the transcript abundance of genes involved in the ICE-CBF-COR cascade dynamically changed in response to cold stress (Figure 2D), and the results revealed a transcriptional regulatory network with candidate genes involved in the cold tolerance in HtKc.

### 3.3. Cold Stress Down-Regulated Photosynthetic Genes in HtKc

**Figure 3:**
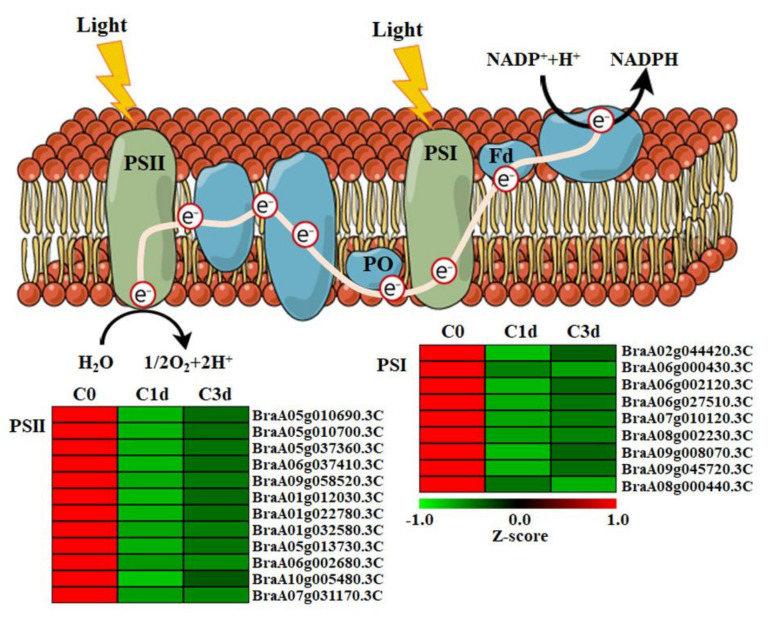
Effects of cold stress on the transcription levels of genes involved in the light reactions. C0, plants grown at 20 °C (control plants); C1d and C3d, plants subjected to cold stress (10 °C) for 1 day and 3 days, respectively. Red and green colors indicate ascending and descending Z-scores, respectively.

When plants are exposed to cold stress, they exhibit several physiological amendments correlated with decreased photosynthetic efficiency [32,33]. In cold-stressed HtKc, we found that several genes involved in the light reactions, Calvin cycle, or photorespiration were differentially expressed, based on the MapMan analysis using all the identified DEGs in the two comparison groups (Figure S1). PSII and PSI are the reaction-center complexes that drive the light reactions of photosynthesis. PSII biogenesis involves the concerted assembly of >20 different polypeptides and a multitude of cofactors, whereas PSI is a multi-subunit protein complex found in thylakoid membranes of chloroplasts [34,35]. Under cold stress, the damage of photoinhibition depends on the rate of the de novo assembly of all subunits and cofactors in PSII [36], indicating that the down-regulation of photosynthetic genes appears to reduce photosynthesis [37]. Regarding the light reaction, 12 and 9 DEGs involved in PSII and PSI, respectively, were detected in cold-stressed HtKc. Noticeably, all the detected DEGs were down-regulated by cold stress (Figure 3). To validate the transcription pattern obtained from the transcriptome analysis, the transcription levels of seven genes involved in PSI or PSII were determined using qRT-PCR. The expression patterns of these genes were found to vary in response to cold stress (Figure S2), indicating that the decreased photosynthesis in cold-stressed HtKc is mediated through the down-regulation of genes involved in PSII and PSI.

### 3.4. Effects of Cold Stress on the Enzymatic Antioxidant Responses

**Figure 4:**
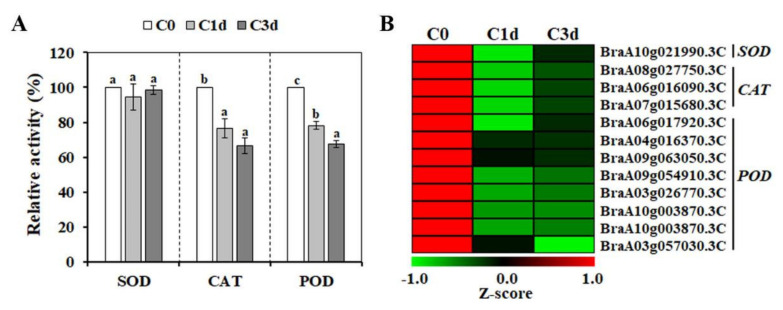
Effect of cold stress on the activity of antioxidant enzymes (**A**) and cold-induced differentially expressed genes encoding antioxidant enzymes (**B**). The data are presented as mean ± SE of five independent experiments. Values with different letters are significantly different, according to Duncan’s multiple range test. Red and green colors indicate ascending and descending Z-scores, respectively. C0, plants grown at 20 °C (control plants); C1d and C3d, plants subjected to cold stress (10 °C) for 1 day and 3 days, respectively.

As described above, cold-induced-ROS accumulation causes severe oxidative damage in plants [2]. Therefore, the induction of antioxidant systems, including enzymatic and non-enzymatic components, has been widely reported as an essential mechanism to control ROS accumulation under environmental-stress conditions [38]. Thus, we analyzed the effect of cold treatment on the activities of enzymatic-antioxidant components. As shown in Figure 4A, a significant decrease in POD and CAT activities was observed with an increased duration of cold stress, whereas the level of SOD activity was not significantly modified.

In higher plants, genotypic variation within a species influences the activation pattern of enzymatic antioxidant components [39,40,41,42,43]. For example, a cold-tolerant banana cultivar exhibits a significantly increased POD activity under cold stress, whereas a cold-sensitive cultivar exhibits a significantly decreased activity [39]. Genes encoding enzymatic antioxidant components display various expression patterns under abiotic stresses [44,45,46,47], implying redundant functions among the members of the same protein family. In the *B*. *rapa* genome, genes encoding 18 *SODs*, 7 *CATs*, and > 140 *PODs* have been identified [44,47]. Among them, 1 *SOD*, 3 *CATs*, and 8 *PODs* were identified as cold-induced DEGs in HtKc, and all the detected DEGs were down-regulated by cold stress (Figure 4B). This observation indicates that the decreased activity of POD or CAT in cold-stressed HtKc might be mediated through the down-regulation of genes encoding PODs or CATs.

### 3.5. Effects of Cold Stress on the Non-Enzymatic Antioxidant Responses

**Figure 5:**
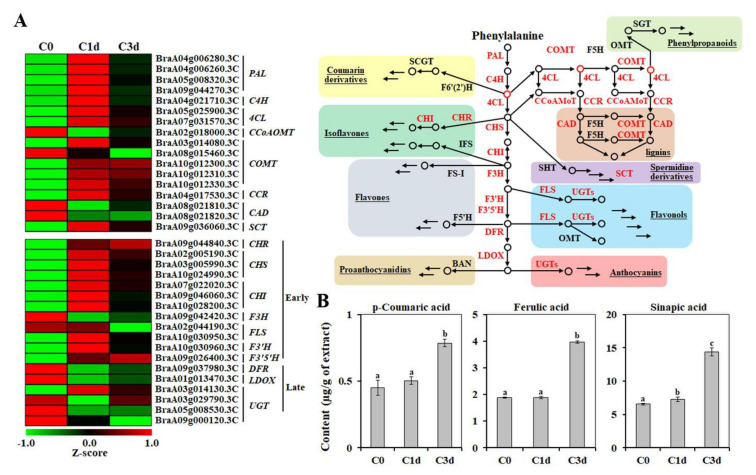
Effect of cold stress on the phenylpropanoid pathway. (**A**) Cold-induced differentially expressed genes involved in the phenylpropanoid pathway. (**B**) The levels of *p*-coumaric, ferulic, and sinapic acids were assessed using HPLC. The data are presented as mean ± SE of five independent experiments. Values with different letters are significantly different, according to Duncan’s multiple range test. C0, plants grown at 20 °C (control plants); C1d and C3d, plants treated cold stress (10 °C) for 1 day and 3 days, respectively. PAL, phenylalanine ammonia-lyase; C4H, cinnamate 4-hydroxylase; 4CL, 4-coumarate:CoA ligase; CCoAOMT, caffeoyl CoA 3-*O*-methyltransferase; COMT, caffeate/5-hydroxyferulate 3-*O*-methyltransferase; CCR, cinnamoyl-CoA reductase; CAD, cinnamyl alcohol dehydrogenase; SCT, sinapoylglucose:choline sinapoyltransferase; CHR, chalcone reductase; CHS, chalcone synthase; CHI, chalcone isomerase; F3H, flavanone 3-hydroxylase; F3′H, flavonoid 3′-hydroxylase; F3′5′H, flavonoid 3′5′-hydroxylase; FLS, flavonol synthase; DFR, dihydroflavonol reductase; LDOX, leucoanthocyanidin dioxygenase; UGT, UDP-glucuronosyl/glucosyl transferase.

In various plants, induction of the antioxidant mechanism decreases ROS levels and triggers tolerance to cold stress [48,49,50]. However, no correlation has been reported between freezing tolerance and the activity of antioxidant enzymes in natural accessions of *Arabidopsis thaliana* [40]. In chickpea, a comparison of enzymatic antioxidant activities, as well as expression levels of related genes between cold-sensitive and cold-tolerant genotypes, has suggested that enzymatic antioxidants are most likely not the major players in cold-stress tolerance [43]. This observation indicates the importance of non-enzymatic antioxidant components, including tocopherol, ascorbate, terpenes, and phenylpropanoids, in response to cold stress [51]. The MapMan analysis revealed that the cold-induced DEGs were distributed in the non-enzymatic antioxidant pathways, including “Ascorbate”, “Glutathione”, “Terpenes”, “Flavonoids”, and “Phenylpropanoids and Phenolics” (Figure S1). Various DEGs involved in the “Terpenes” pathway were down-regulated, whereas DEGs in the “Phenylpropanoids and Phenolics” pathway were up-regulated by cold stress (Figure S1 and Figure 5A). Regarding the “Flavonoids” pathway, a large number of DEGs involved in the early flavonoid pathway were up-regulated, whereas those in the late flavonoid pathway were down-regulated (Figure 5A). Taken together, this observation indicated that polyphenolic compounds related to the phenylpropanoid pathway are important metabolites in cold-stressed HtKc. To investigate whether the up-regulation of these genes is related to the accumulation of polyphenolic compounds, we assessed cold-induced variation in the content of *p*-coumaric, ferulic, and sinapic acids, which are catalyzed by cinnamate 4-hydroxylase (C4H) and caffeic acid *O*-methyltransferase (COMT), respectively. As shown in Figure 5B, the levels of *p*-coumaric acid (0.45 ± 0.05 µg/g–0.78 ± 0.02 µg/g of extract), ferulic acid (1.88 ± 0.02 µg/g–3.96 ± 0.04 µg/g of extract), and sinapic acid (6.55 ± 0.12 µg/g–14.38 ± 0.57 µg/g of extract) in the leaves of HtKc were increased in response to cold stress, indicating that the accumulation of these compounds was due to increased expression of phenylpropanoid biosynthetic genes in response to cold stress. Similar to our findings, thermal stress induces the biosynthesis of phenolics while inhibiting their oxidation, thereby causing their accumulation, in tomato and watermelon plants [52].

**Figure 6:**
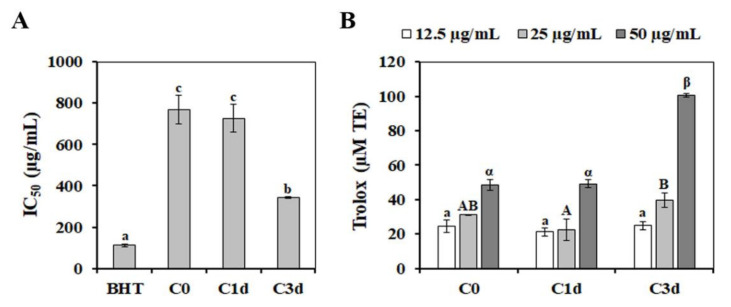
Chemical-based antioxidant activity was measured using the DPPH-radical-scavenging (**A**) and ORAC (**B**) assays. The DPPH-radical-scavenging activity and ORAC values are expressed as IC_50_ and μM of Trolox equivalents, respectively. The data are presented as mean ± SE of five independent experiments. Values with different letters are significantly different, according to Duncan’s multiple range test. C0, plants grown at 20 °C (control plants); C1d and C3d, plants treated cold stress (10 °C) for 1 day and 3 days, respectively.

Polyphenolic compounds are well known as free-radical scavengers and have therapeutic potentials against diseases resulting from oxidative stress [53]. Therefore, we presumed that the induction of the phenylpropanoid pathway is required for enhancing the antioxidant activity in response to cold stress. To test this hypothesis, the free-radical scavenging activity and oxygen-radical antioxidant capacity of MeOH extracts of cold-stressed HtKc plants were assessed. As shown in Figure 6A, C3d extract (IC_50_ = 342.82 ± 5.18 µg/mL) showed the highest DPPH-free radical scavenging activity, followed by C1d extract (IC_50_ = 726.39 ± 68.27 µg/mL). Similarly, based on the ORAC assay, the highest antioxidant capacity was observed in the C3d extract (Figure 6B), suggesting that the phenylpropanoids pathway plays an essential role in the regulation of resistance to cold-induced ROS in HtKc. In the peanut seedlings, the accumulation of polyphenolic compounds via the phenylpropanoid pathway has been suggested to increase the selenite tolerance [54]. In addition, metabolites related to the phenylpropanoid pathway play important roles in the regulation of resistance to drought stress in the foxtail millet [55]. Furthermore, polyphenolics, including flavonoids, are determinants of the freezing tolerance in *Arabidopsis thaliana* [56]. These observations indicate that the phenylpropanoid pathway is a potential target for improving plant tolerance against environmental stresses.

## 4. Conclusions

Understanding the molecular mechanism whereby crops respond to cold stress is an important step for increasing the cold tolerance of plants. This study provides an overview of the molecular changes in HtKc upon cold stress. The cold-induced DEGs exhibit differential expression patterns of antioxidant components. In addition, the analysis of chemical-based antioxidant activity revealed that the accumulation of polyphenolic compounds, which have been proven to be responsible for eradicating cold-induced ROS in HtKc, through induction of the phenylpropanoid pathway is presumably the major cold-stress response in this plant. Therefore, future studies should more deeply investigate the functional application of polyphenolic compounds to increase cold tolerance. We strongly believe that our data provide a solid foundation for future studies to understand the biochemical and molecular mechanisms underlying the response to cold stress in HtKc.

## Data Availability

The data presented in this study are available on request from the corresponding author. The data are not publicly available due to reasons of privacy.

## Supplementary Materials

The following supporting information are available online at: https://www.mdpi.com/article/10.3390/antiox11040700/s1, Figure S1: MapMan-based overview of the metabolism-related differentially expressed genes in each comparison; Figure S2: The transcription levels of the selected genes involved in the light reactions were determined using qPCR analysis; Table S1: Primer sequences used for the qPCR analysis; Table S2: Summary of the RNA-seq data from the three RNA libraries derived from the control and cold-stressed leaf groups each; Table S3. Top 100 up- and down-regulated DEGs in cold-treated heading type Kimchi Cabbage.

## References

[1] Ding Y., Shi Y., Yang S. (2019). Advances and challenges in uncovering cold tolerance regulatory mechanisms in plants. New Phytol..

[2] Liang S.-M., Kuang J.-F., Ji S.-J., Chen Q.-F., Deng W., Min T., Shan W., Chen J.-Y., Lu W.-J. (2020). The membrane lipid metabolism in horticultural products suffering chilling injury. Food Qual. Saf..

[3] Ngaffo Mekontso F., Duan W., Cisse E.H.M., Chen T., Xu X. (2021). Alleviation of postharvest chilling injury of carambola fruit by γ-aminobutyric acid: Physiological, biochemical, and structural characterization. Front. Nutr..

[4] Shi J., Zuo J., Xu D., Gao L., Wang Q. (2019). Effect of low-temperature conditioning combined with methyl jasmonate treatment on the chilling resistance of eggplant (*Solanum melongena* L.) fruit. J. Food Sci. Technol..

[5] Lin K.H., Sei S.C., Su Y.H. (2019). Chiang CM. Overexpression of the Arabidopsis and winter squash superoxide dismutase genes enhances chilling tolerance via ABA-sensitive transcriptional regulation in transgenic Arabidopsis. Plant. Signal. Behav..

[6] Lee H.J., Kim J.S., Lee S.G., Kim S.K., Min B., Choi C.S. (2017). Glutamic acid foliar application enhances antioxidant enzyme activities in kimchi cabbages leaves treated with low air temperature. Hortic. Sci. Technol..

[7] Sim H.S., Jo W.J., Lee H.J., Moon Y.H., Woo U.J., Jung S.B., Ahn S.R., Kim S.K. (2021). Determination of optimal growing degree days and cultivars of kimchi cabbage for growth and yield during spring cultivation under shading conditions. Hortic. Sci. Technol..

[8] Fan H., Du C., Xu Y., Wu X. (2014). Exogenous nitric oxide improves chilling tolerance of Chinese cabbage seedlings by affecting antioxidant enzymes in leaves. Hort. Environ. Biotechnol..

[9] Thamilarasan S.K., Park J.-I., Jung H.-J., Chung M.-Y., Cho Y.-G., Nou I.-S. (2016). Expression profiling and characterization of cold, freezing-related genes from Brassica rapa cultivars. Isr. J. Plant Sci..

[10] Yu J.-G., Lee G.-H., Lee S.-C., Park Y.-D. (2014). Gene expression and phenotypic analyses of transgenic Chinese cabbage over-expressing the cold tolerance gene, BrCSR. Hort. Environ. Biotechnol..

[11] Xiang D., Chai Y., Man L., Sun Y., Zhang T., Wei C., Xie Z., Li H., Zhang W., Liu D. (2017). Overexpression of a heading Chinese cabbage ICE1 gene confers freezing tolerance in transgenic rice. Plant. Cell Tiss. Org..

[12] Wang W., Wu P., Li Y., Hou X. (2016). Genome-wide analysis and expression patterns of ZF-HD transcription factors under different developmental tissues and abiotic stresses in Chinese cabbage. Mol. Genet. Genom..

[13] Huang F., Wang J., Tang J., Hou X. (2019). Identification, evolution and functional inference on the cold-shock domain protein family in Pak-choi (*Brassica rapa* ssp. chinensis) and Chinese cabbage (*Brassica rapa* ssp. pekinensis). J. Plant Interact..

[14] Huang F., Wang J., Duan W., Hou X. (2020). Identification and expression analysis of cold shock protein 3 (BcCSP3) in non-heading Chinese Cabbage (*Brassica rapa* ssp. chinensis). Plants.

[15] Kayum M.A., Park J.I., Nath U.K., Saha G., Biswas M.K., Kim H.T., Nou I.S. (2017). Genome-wide characterization and expression profiling of PDI family gene reveals function as abiotic and biotic stress tolerance in Chinese cabbage (*Brassica rapa* ssp. pekinensis). BMC Genom..

[16] Bates L.S., Waldren R.P., Teare I.D. (1973). Rapid determination of free proline for water-stress studies. Plant Soil.

[17] Bradford M.M. (1976). A rapid and sensitive method for the quantitation of microgram quantities of protein utilizing the principle of protein-dye binding. Anal. Biochem..

[18] Choi J.H., Kim H., Hyun T.K. (2018). Transcriptome analysis of *Abeliophyllum distichum* NAKAI reveals potential molecular markers and candidate genes involved in anthocyanin biosynthesis pathway. S. Afr. J. Bot..

[19] Eom S.H., Baek S.A., Kim J.K., Hyun T.K. (2018). Transcriptome analysis in Chinese cabbage (*Brassica rapa* ssp. pekinensis) provides the role of glucosinolate metabolism in response to drought stress. Molecules.

[20] Hong C.P., Kim J., Lee J., Yoo S.I., Bae W., Geem K.R., Yu J., Jang I., Jo I.H., Cho H. (2021). Gibberellin signaling promotes the secondary growth of storage roots in Panax ginseng. Int. J. Mol. Sci..

[21] Kim E., Mok H.K., Hyun T.K. (2022). Variations in the antioxidant, anticancer, and anti-inflammatory properties of different Rosa rugosa organ extracts. Agronomy.

[22] Kenchanmane Raju S.K., Barnes A.C., Schnable J.C., Roston R.L. (2018). Low-temperature tolerance in land plants: Are transcript and membrane responses conserved?. Plant. Sci..

[23] Verslues P.E., Sharma S. (2010). Proline metabolism and its implications for plant-environment interaction. Arab. Book.

[24] Hayat S., Hayat Q., Alyemeni M.N., Wani A.S., Pichtel J., Ahmad A. (2012). Role of proline under changing environments: A review. Plant. Signal Behav..

[25] Wei Y., Chen H., Wang L., Zhao Q., Wang D., Zhang T. (2021). Cold acclimation alleviates cold stress-induced PSII inhibition and oxidative damage in tobacco leaves. Plant. Signal. Behav..

[26] Jian H., Xie L., Wang Y., Cao Y., Wan M., Lv D., Li J., Lu K., Xu X., Liu L. (2020). Characterization of cold stress responses in different rapeseed ecotypes based on metabolomics and transcriptomics analyses. PeerJ.

[27] Pu Y., Liu L., Wu J., Zhao Y., Bai J., Ma L., Yue J., Jin J., Niu Z., Fang Y. (2019). Transcriptome profile analysis of winter rapeseed (*Brassica napus* L.) in response to freezing stress, reveal potentially connected events to freezing stress. Int. J. Mol. Sci..

[28] Chen H., Chen X., Chen D., Li J., Zhang Y., Wang A. (2015). A comparison of the low temperature transcriptomes of two tomato genotypes that differ in freezing tolerance: *Solanum lycopersicum* and *Solanum habrochaites*. BMC Plant. Biol..

[29] Jin J., Zhang H., Zhang J., Liu P., Chen X., Li Z., Xu Y., Lu P., Cao P. (2017). Integrated transcriptomics and metabolomics analysis to characterize cold stress responses in *Nicotiana tabacum*. BMC Genom..

[30] Meng D., Yu X., Ma L., Hu J., Liang Y., Liu X., Yin H., Liu H., He X., Li D. (2017). Transcriptomic response of Chinese yew (*Taxus chinensis*) to cold stress. Front. Plant Sci..

[31] Hwarari D., Guan Y., Ahmad B., Movahedi A., Min T., Hao Z., Lu Y., Chen J., Yang L. (2022). ICE-CBF-COR signaling cascade and its regulation in plants responding to cold stress. Int. J. Mol. Sci..

[32] Hajihashemi S., Noedoost F., Geuns J.M.C., Djalovic I., Siddique K.H.M. (2018). Effect of cold stress on photosynthetic traits, carbohydrates, morphology, and anatomy in nine cultivars of *Stevia rebaudiana*. Front. Plant Sci..

[33] Savitch L.V., Ivanov A.G., Gudynaite-Savitch L., Huner N.P., Simmonds J. (2011). Cold stress effects on PSI photochemistry in Zea mays: Differential increase of FQR-dependent cyclic electron flow and functional implications. Plant. Cell Physiol..

[34] Nickelsen J., Rengstl B. (2013). Photosystem II assembly: From cyanobacteria to plants. Annu. Rev. Plant Biol..

[35] Jensen P.E., Bassi R., Boekema E.J., Dekker J.P., Jansson S., Leister D., Robinson C., Scheller H.V. (2007). Structure, function and regulation of plant photosystem I. Biochim. Biophys. Acta.

[36] Zhuang K., Kong F., Zhang S., Meng C., Yang M., Liu Z., Wang Y., Ma N., Meng Q. (2019). Whirly1 enhances tolerance to chilling stress in tomato via protection of photosystem II and regulation of starch degradation. New Phytol..

[37] Chinnusamy V., Zhu J.K., Sunkar R. (2010). Gene regulation during cold stress acclimation in plants. Methods Mol. Biol..

[38] Singh R., Singh S., Parihar P., Mishra R.K., Tripathi D.K., Singh V.P., Chauhanm D.K., Prasad S.M. (2016). Reactive oxygen species (ROS): Beneficial companions of plants’ developmental processes. Front. Plant. Sci..

[39] Zhang Q., Zhang J.Z., Chow W.S., Sun L.L., Chen J.W., Chen Y.J., Peng C.L. (2011). The influence of low temperature on photosynthesis and antioxidant enzymes in sensitive banana and tolerant plantain (*Musa* sp.) cultivars. Photosynthetica.

[40] Distelbarth H., Nägele T., Heyer A.G. (2013). Responses of antioxidant enzymes to cold and high light are not correlated to freezing tolerance in natural accessions of *Arabidopsis thaliana*. Plant. Biol..

[41] Niu Y., Liu Z., He H., Han X., Qi Z., Yang Y. (2020). Gene expression and metabolic changes of *Momordica charantia* L. seedlings in response to low temperature stress. PLoS ONE.

[42] Dey S., Biswas A., Huang S., Li D., Liu L., Deng Y., Xiao A., Birhanie Z.M., Zhang J., Li J. (2021). Low temperature effect on different varieties of *Corchorus capsularis* and *Corchorus olitorius* at seedling stage. Agronomy.

[43] Kiran A., Sharma P.N., Awasthi R., Nayyar H., Seth R., Chandel S.S., Siddique K.H.M., Zinta G., Sharma K.D. (2021). Disruption of carbohydrate and proline metabolism in anthers under low temperature causes pollen sterility in chickpea. Environ. Exp. Bot..

[44] Verma D., Lakhanpal N., Singh K. (2019). Genome-wide identification and characterization of abiotic-stress responsive SOD (*superoxide dismutase*) gene family in *Brassica juncea* and *B. rapa*. BMC Genom..

[45] Cai K., Liu H., Chen S., Liu Y., Zhao X., Chen S. (2021). Genome-wide identification and analysis of class III peroxidases in *Betula pendula*. BMC Genom..

[46] Aleem M., Riaz A., Raza Q., Aleem M., Aslam M., Kong K., Atif R.M., Kashif M., Bhat J.A., Zhao T. (2022). Genome-wide characterization and functional analysis of class III peroxidase gene family in soybean reveal regulatory roles of GsPOD40 in drought tolerance. Genomics.

[47] Raza A., Su W., Gao A., Mehmood S.S., Hussain M.A., Nie W., Lv Y., Zou X., Zhang X. (2021). Catalase (CAT) gene family in rapeseed (*Brassica napus* L.): Genome-wide analysis, identification, and expression pattern in response to multiple hormones and abiotic stress conditions. Int. J. Mol. Sci..

[48] Lado J., Rodrigo M.J., Cronje P., Zacarías L. (2015). Involvement of lycopene in the induction of tolerance to chilling injury in grapefruit. Posthar. Biol. Technol..

[49] Vighi I.L., Benitez L.C., Amaral M.N., Moraes G.P., Auler P.A., Rodrigues G.S., Deuner S., Maia L.C., Braga E.J.B. (2017). Functional characterization of the antioxidant enzymes in rice plants exposed to salinity stress. Biol. Plant..

[50] De Araújo N.O., Santos M.N.D.S., de Araujo F.F., Véras M.L.M., Tello J.P.D.J., Arruda R.D.S., Fugate K.K., Finger F.L. (2021). Balance between oxidative stress and the antioxidant system is associated with the level of cold tolerance in sweet potato roots. Postharvest Biol. Technol..

[51] Hasanuzzaman M., Bhuyan M.H.M.B., Zulfiqar F., Raza A., Mohsin S.M., Mahmud J.A., Fujita M., Fotopoulos V. (2020). Reactive oxygen species and antioxidant defense in plants under abiotic stress: Revisiting the crucial role of a universal defense regulator. Antioxidants.

[52] Rivero R.M., Ruiz J.M., García P.C., López-Lefebre L.R., Sánchez E., Romero L. (2001). Resistance to cold and heat stress: Accumulation of phenolic compounds in tomato and watermelon plants. Plant. Sci..

[53] Lv Q.-Z., Long J.-T., Gong Z.-F., Nong K.-Y., Liang X.-M., Qin T., Huang W., Yang L. (2021). Current state of knowledge on the antioxidant effects and mechanisms of action of polyphenolic compounds. Nat. Prod. Commun..

[54] Wang G., Wu L., Zhang H., Wu W., Zhang M., Li X., Wu H. (2016). Regulation of the phenylpropanoid pathway: A mechanism of selenium tolerance in peanut (*Arachis hypogaea* L.) seedlings. J. Agric. Food Chem..

[55] Yu A., Zhao J., Wang Z., Cheng K., Zhang P., Tian G., Liu X., Guo E., Du Y., Wang Y. (2020). Transcriptome and metabolite analysis reveal the drought tolerance of foxtail millet significantly correlated with phenylpropanoids-related pathways during germination process under PEG stress. BMC Plant. Biol..

[56] Schulz E., Tohge T., Zuther E., Fernie A.R., Hincha D.K. (2016). Flavonoids are determinants of freezing tolerance and cold acclimation in *Arabidopsis thaliana*. Sci. Rep..

