# Supplementary material for: Plant Response to Cold Stress: Cold Stress Changes Antioxidant Metabolism in Heading Type Kimchi Cabbage (Brassica rapa L. ssp. Pekinensis)
Source: Antioxidants (Basel). 2022 Apr 1;11(4):700. doi: 10.3390/antiox11040700 (PMC9031148; doi:10.3390/antiox11040700)
Supplement: Supplementary file 1 [file antioxidants-11-00700-s001.zip › antioxidants-1654508-supplementary.pdf]

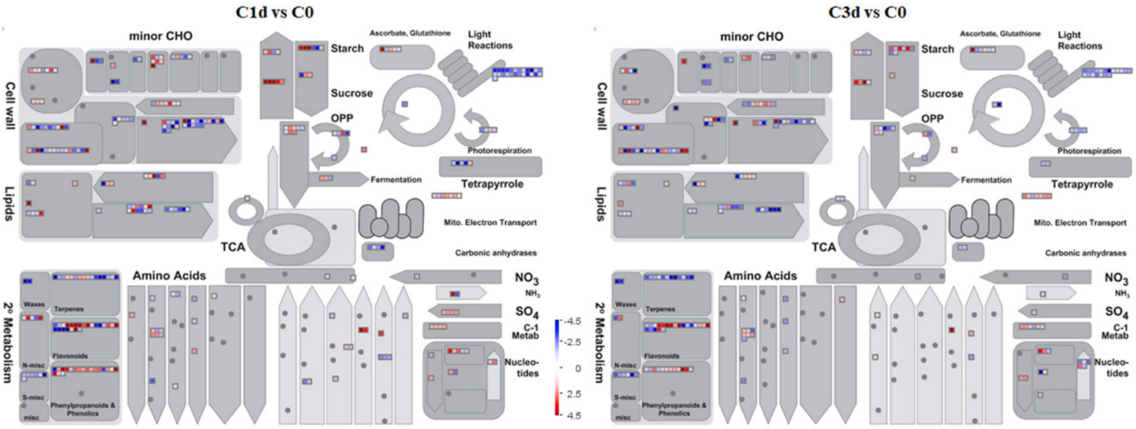

Figure S1. MapMan-based overview of the metabolism-related differentially expressed genes in each comparison.

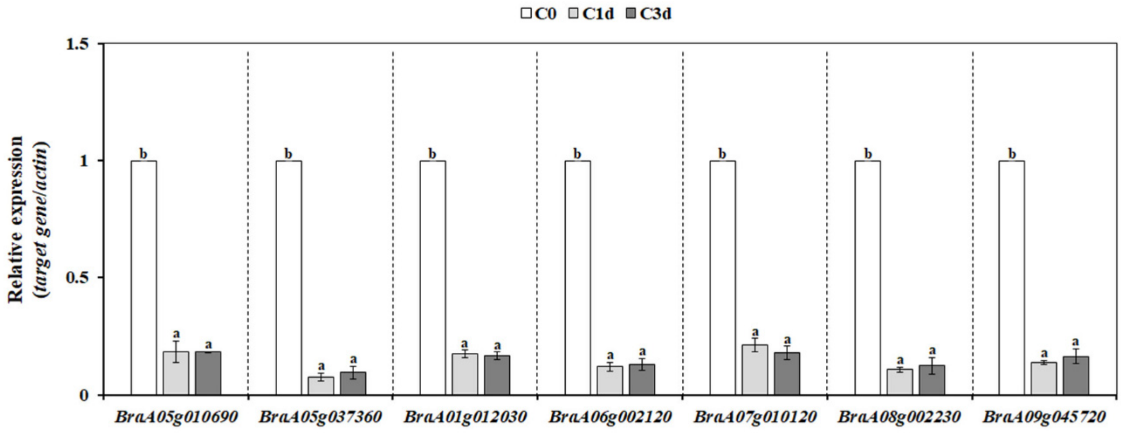

Figure S2. The transcription levels of the selected genes involved in the light reactions were determined using qPCR analysis. Values with different letters are significantly different, according to Duncan's multiple range test.

Table S1. Primer sequences used for qPCR analysis.

Primer	Sequences (5`-3`)	Accession number
BrCRG2-F	CAAGTCACCGAGCATCATCTG	Bra032023
BrCRG2-Rev	CGTAACCGCCAGAGAACTTC	
BrCRG3-F	ACCACTTCACCGAAGCAATC	Bra036648
BrCRG3-Rev	GCTTCTTCGAGCTGGTTCAG	
BrCRG5-F	ATTGCGTTCAAGAGGGTAGTG	Bra009356
BrCRG5-Rev	ATGGATCTCTCTGACGTTCAG	
BraA05g010690-F	GCTTTCGCAGAGCTGAAGGT	BraA05g010690.3C
BraA05g010690-Rev	CCCTTTCCAGTGACGATGGC	
BraA05g037360-F	CGCTGCTGCTTCTTCCATCA	BraA05g037360.3C
BraA05g037360-Rev	ATCCAAACCGGGCTGTGAAC	
BraA01g012030-F	GACGGTGCCTCAAAGGTCTG	BraA01g012030.3C
BraA01g012030-Rev	GCGAGGACAGCCTGAACAAA	
BraA05g013730-F	TCCCTTCACCACGTGTCCTT	BraA05g013730.3C
BraA05g013730-Rev	ACGGGAGATGGCGGAGTTAG	
BraA06g002120-F	TACTCCGCCGTGAAAGGACT	BraA06g002120.3C
BraA06g002120-Rev	AGCTTTGGCGAGAAGGCTTG	
BraA07g010120-F	GACGCAGCAACATCAGTCCA	BraA07g010120.3C
BraA07g010120-Rev	CGGAGCTGAGAGGAGGATGG	
BraA08g002230-F	CTGCCACCGTCAAAGGACTC	BraA08g002230.3C
BraA08g002230-Rev	GGCACCAGCTCTTGTGGATT	
BraA09g045720-F	GCAGATGGGCTATGCTTGCT	BraA09g045720.3C
BraA09g045720-Rev	ACTGCTGCCCATTCTTGAGC	
Actin-F	TGGGCGTACTACTGGTATTGTG	JN120480.1
Actin-Rev	TGTAACCTCTCTCGGTGAGAAT	

Table S2. Summary of RNA sequencing data from 3 RNA libraries of control and cold-stressed leaves.

Sample No.	Cold stress	Clean reads	Clean bases (Gb)	Mapped reads (%)	Accession number (NABIC)
C0	Non-treated	33,638,792	3.16	94.03	NN-7988
C1d	1 day	33,125,312	3.12	94.48	NN-7986
C3d	3 days	35,788,312	3.34	93.51	NN-7987

Table S3. Top 100 up- and down-regulated DEGs in cold-treated heading type Kimchi Cabbage.

C1d vs C0			C3d vs C0		
**Gene id**	**log2FoldChange**	**Regulation**	**Gene id**	**log2FoldChange**	**Regulation**
BraA03g035360.3C	8.144813501	UP	BraA03g022330.3C	7.259010921	UP
BraA10g030360.3C	7.856398429	UP	BraA02g012200.3C	7.076512231	UP
BraA01g031210.3C	7.757891171	UP	BraA03g003870.3C	7.010757785	UP
BraA08g029150.3C	7.664096238	UP	BraA10g023640.3C	6.884488067	UP
BraA03g003870.3C	7.619530685	UP	BraA03g049670.3C	6.694425153	UP
BraA10g023640.3C	7.451172979	UP	BraA10g030360.3C	6.578172085	UP
BraA03g005990.3C	7.377557895	UP	BraA03g005990.3C	6.326693369	UP
BraA03g022330.3C	7.059685768	UP	BraA03g022340.3C	6.17265217	UP
BraA03g022340.3C	6.623018828	UP	BraA06g005750.3C	5.768951727	UP
BraA02g012200.3C	6.495541968	UP	BraA01g031350.3C	5.673241863	UP
BraA09g054400.3C	6.333193645	UP	BraA07g010270.3C	5.666228261	UP
BraA06g005750.3C	6.200963103	UP	BraA05g024380.3C	5.630764826	UP
BraA01g031350.3C	6.157040008	UP	BraA09g054400.3C	5.62868219	UP
BraA05g024380.3C	6.152370912	UP	BraA07g020170.3C	5.627754114	UP
BraA10g003120.3C	6.147693394	UP	BraA03g040550.3C	5.35469184	UP
BraA02g006880.3C	6.140877087	UP	BraA07g000330.3C	5.337163985	UP
BraA03g049670.3C	6.068120744	UP	BraA02g006290.3C	5.312015955	UP
BraA05g041980.3C	5.965027251	UP	BraA08g032010.3C	5.2995047	UP
BraA09g046060.3C	5.951107274	UP	BraA01g031210.3C	5.276281743	UP
BraA09g013640.3C	5.853995939	UP	BraA09g013320.3C	5.276281743	UP
BraA03g040550.3C	5.849265598	UP	BraA03g035360.3C	5.251190762	UP
BraA09g046410.3C	5.71617178	UP	BraA08g022980.3C	5.199660461	UP
BraA03g039140.3C	5.701992845	UP	BraA02g006880.3C	5.132546266	UP
BraA08g022980.3C	5.691266439	UP	BraA02g038610.3C	5.118740466	UP
BraA08g032010.3C	5.661835719	UP	BraA02g022400.3C	5.062156938	UP
BraA04g000750.3C	5.579373559	UP	BraA07g013580.3C	5.062156938	UP
BraA05g039490.3C	5.532067844	UP	BraA10g003470.3C	5.062156938	UP
BraA02g006290.3C	5.473515662	UP	BraA10g003120.3C	5.060232061	UP
BraA04g016370.3C	5.353097703	UP	BraA04g016370.3C	5.003263249	UP
BraA08g026920.3C	5.353097703	UP	BraA06g044080.3C	4.990685183	UP
BraA05g039480.3C	5.349312093	UP	BraA07g022330.3C	4.9728896	UP
BraA09g004550.3C	5.21613849	UP	BraA09g001840.3C	4.960558798	UP
BraA03g011160.3C	5.210139749	UP	BraA07g023060.3C	4.877732367	UP
BraA09g035960.3C	5.195032857	UP	BraA09g000560.3C	4.877732367	UP
BraA09g014500.3C	5.1797661	UP	BraA03g052550.3C	4.873627968	UP
BraA05g029790.3C	5.148739204	UP	BraA01g023310.3C	4.844565503	UP
BraA07g020170.3C	5.148739204	UP	BraA04g019150.3C	4.844565503	UP
BraA09g001840.3C	5.123428116	UP	BraA08g026920.3C	4.844565503	UP
BraA01g029770.3C	5.117030345	UP	BraA03g031220.3C	4.775852753	UP
BraA07g008050.3C	5.117030345	UP	BraA07g036610.3C	4.775852753	UP
BraA10g004100.3C	5.117030345	UP	BraA06g040090.3C	4.740228843	UP
BraA10g028200.3C	5.10697668	UP	BraA07g041010.3C	4.740228843	UP
BraA10g024990.3C	5.099878209	UP	BraA09g014500.3C	4.740228843	UP
BraA09g003840.3C	5.084608867	UP	BraA09g065180.3C	4.740228843	UP
BraA07g014330.3C	5.078036213	UP	BraA05g015310.3C	4.718424473	UP
BraA01g004620.3C	5.051442003	UP	BraA03g017290.3C	4.703702967	UP
BraA03g027800.3C	5.051442003	UP	BraA08g029150.3C	4.703702967	UP
BraA03g060300.3C	5.051442003	UP	BraA09g003840.3C	4.703702967	UP
BraA07g011910.3C	5.051442003	UP	BraA05g005380.3C	4.666228261	UP
BraA10g003470.3C	5.051442003	UP	BraA07g008050.3C	4.666228261	UP
BraA03g052150.3C	4.982729253	UP	BraA07g042530.3C	4.666228261	UP
BraA04g027910.3C	4.982729253	UP	BraA05g039480.3C	4.663060982	UP
BraA09g062410.3C	4.966214166	UP	BraA02g035830.3C	4.643267113	UP
BraA02g008800.3C	4.966117312	UP	BraA05g041980.3C	4.627754114	UP
BraA03g047370.3C	4.958123361	UP	BraA06g011010.3C	4.627754114	UP
BraA07g042530.3C	4.947105343	UP	BraA09g045650.3C	4.627754114	UP
BraA09g033560.3C	4.947105343	UP	BraA07g041990.3C	4.614697961	UP
BraA01g015290.3C	4.873104762	UP	BraA04g032510.3C	4.608125307	UP
BraA04g030410.3C	4.873104762	UP	BraA09g046060.3C	4.590450414	UP
BraA02g006530.3C	4.834630614	UP	BraA08g022730.3C	4.588225749	UP
BraA03g026740.3C	4.830291678	UP	BraA09g013640.3C	4.588225749	UP

BraA05g018320.3C	4.79510225	UP	BraA10g012300.3C	4.492918789	UP
BraA09g033530.3C	4.79510225	UP	BraA09g033560.3C	4.462694867	UP
BraA02g015440.3C	4.790681954	UP	BraA01g004620.3C	4.418300748	UP
BraA02g013880.3C	4.781681734	UP	BraA10g022220.3C	4.395580671	UP
BraA06g022370.3C	4.774924368	UP	BraA04g030410.3C	4.325191344	UP
BraA06g029570.3C	4.773075943	UP	BraA01g032400.3C	4.251190762	UP
BraA09g007710.3C	4.754460265	UP	BraA04g000750.3C	4.251190762	UP
BraA10g020570.3C	4.754460265	UP	BraA07g005550.3C	4.242729183	UP
BraA05g005380.3C	4.71264009	UP	BraA08g024770.3C	4.242729183	UP
BraA06g040090.3C	4.71264009	UP	BraA03g058630.3C	4.199660461	UP
BraA10g012300.3C	4.693918723	UP	BraA10g024990.3C	4.196414802	UP
BraA07g023060.3C	4.691266439	UP	BraA02g044900.3C	4.17318825	UP
BraA08g026200.3C	4.65860025	UP	BraA03g006910.3C	4.17318825	UP
BraA10g033680.3C	4.651977308	UP	BraA03g009710.3C	4.17318825	UP
BraA03g031220.3C	4.625177248	UP	BraA06g010490.3C	4.118740466	UP
BraA07g010270.3C	4.625177248	UP	BraA02g006530.3C	4.104801275	UP
BraA05g015310.3C	4.579373559	UP	BraA03g026420.3C	4.09072609	UP
BraA09g055030.3C	4.56850984	UP	BraA08g001040.3C	4.09072609	UP
BraA03g002690.3C	4.555914586	UP	BraA05g039490.3C	4.062156938	UP
BraA09g000540.3C	4.54678935	UP	BraA03g028610.3C	4.033010592	UP
BraA02g000880.3C	4.532067844	UP	BraA08g016290.3C	4.003263249	UP
BraA03g017290.3C	4.532067844	UP	BraA09g035960.3C	4.003263249	UP
BraA04g019150.3C	4.532067844	UP	BraA09g046410.3C	3.993209584	UP
BraA10g030950.3C	4.530781446	UP	BraA09g046320.3C	3.941862704	UP
BraA02g008810.3C	4.497302426	UP	BraA07g004890.3C	3.917607164	UP
BraA06g000690.3C	4.483158243	UP	BraA10g000690.3C	3.903727575	UP
BraA09g024560.3C	4.483158243	UP	BraA10g026970.3C	3.899427438	UP
BraA07g041010.3C	4.473174155	UP	BraA10g028200.3C	3.881825121	UP
BraA09g038250.3C	4.458067262	UP	BraA02g014340.3C	3.877732367	UP
BraA10g009060.3C	4.458067262	UP	BraA04g027910.3C	3.877732367	UP
BraA01g034810.3C	4.43253217	UP	BraA06g000690.3C	3.844565503	UP
BraA10g000820.3C	4.418538898	UP	BraA01g016020.3C	3.837839639	UP
BraA05g001940.3C	4.373764762	UP	BraA01g035990.3C	3.810618171	UP
BraA08g035760.3C	4.370741971	UP	BraA06g005950.3C	3.810618171	UP
BraA08g034250.3C	4.353097703	UP	BraA06g035270.3C	3.810618171	UP
BraA03g052550.3C	4.325616966	UP	BraA09g000540.3C	3.810618171	UP
BraA07g004890.3C	4.287428444	UP	BraA03g060300.3C	3.775852753	UP
BraA06g039490.3C	4.269033438	UP	BraA10g012310.3C	3.756073121	UP
BraA02g005190.3C	4.233569415	UP	BraA05g035890.3C	3.740228843	UP
BraA02g025470.3C	-8.846497966	Down	BraA05g033670.3C	-7.948021466	Down
BraA02g022450.3C	-8.576456613	Down	BraA04g018050.3C	-6.626093371	Down
BraA09g046950.3C	-7.929411603	Down	BraA02g045650.3C	-6.558979176	Down
BraA09g021230.3C	-7.775702188	Down	BraA05g040740.3C	-6.452063972	Down
BraA03g016290.3C	-7.514374104	Down	BraA03g017510.3C	-6.414589266	Down
BraA08g017250.3C	-6.898384708	Down	BraA03g001030.3C	-6.363058966	Down
BraA07g030850.3C	-6.769999829	Down	BraA07g030360.3C	-6.136288104	Down
BraA09g057600.3C	-6.641609292	Down	BraA09g009300.3C	-6.105261208	Down
BraA02g028660.3C	-6.329019062	Down	BraA09g041760.3C	-6.007964007	Down
BraA04g032140.3C	-6.257465801	Down	BraA05g036120.3C	-5.939251257	Down
BraA08g003730.3C	-6.257465801	Down	BraA09g059710.3C	-5.867101471	Down
BraA03g025730.3C	-6.232803747	Down	BraA05g018870.3C	-5.751624254	Down
BraA01g001220.3C	-6.182177674	Down	BraA09g013070.3C	-5.751624254	Down
BraA09g045780.3C	-6.182177674	Down	BraA02g009810.3C	-5.710982269	Down
BraA10g031990.3C	-6.182177674	Down	BraA02g015610.3C	-5.710982269	Down
BraA09g009510.3C	-5.989532596	Down	BraA05g033000.3C	-5.710982269	Down
BraA07g030360.3C	-5.929411603	Down	BraA03g041150.3C	-5.669162093	Down
BraA01g015000.3C	-5.866675848	Down	BraA01g007420.3C	-5.626093371	Down
BraA10g022800.3C	-5.83425437	Down	BraA03g029050.3C	-5.626093371	Down
BraA09g041760.3C	-5.801087506	Down	BraA01g039860.3C	-5.581699252	Down
BraA04g003690.3C	-5.767140175	Down	BraA03g042980.3C	-5.581699252	Down
BraA02g007790.3C	-5.504105769	Down	BraA07g010670.3C	-5.581699252	Down
BraA02g015610.3C	-5.504105769	Down	BraA08g005150.3C	-5.581699252	Down
BraA03g039860.3C	-5.504105769	Down	BraA02g012590.3C	-5.535895562	Down
BraA01g020190.3C	-5.483347209	Down	BraA09g003600.3C	-5.535895562	Down
BraA04g003650.3C	-5.462285593	Down	BraA09g029330.3C	-5.535895562	Down

BraA02g031060.3C	-5.440911942	Down	BraA06g031860.3C	-5.488589848	Down
BraA03g044630.3C	-5.419216871	Down	BraA07g029340.3C	-5.488589848	Down
BraA02g042520.3C	-5.374822752	Down	BraA09g028180.3C	-5.488589848	Down
BraA03g051090.3C	-5.374822752	Down	BraA07g037480.3C	-5.450301363	Down
BraA05g040580.3C	-5.374822752	Down	BraA02g017260.3C	-5.439680247	Down
BraA02g045650.3C	-5.352102675	Down	BraA03g063330.3C	-5.414589266	Down
BraA05g019900.3C	-5.291299396	Down	BraA07g003040.3C	-5.389054174	Down
BraA02g045640.3C	-5.281713347	Down	BraA03g047100.3C	-5.35588731	Down
BraA05g034370.3C	-5.281713347	Down	BraA01g034560.3C	-5.336586754	Down
BraA06g034760.3C	-5.281713347	Down	BraA03g046150.3C	-5.336586754	Down
BraA07g042830.3C	-5.281713347	Down	BraA07g028680.3C	-5.336586754	Down
BraA06g036860.3C	-5.232803747	Down	BraA07g038350.3C	-5.336586754	Down
BraA02g019700.3C	-5.221706038	Down	BraA01g020460.3C	-5.28213897	Down
BraA05g012850.3C	-5.182177674	Down	BraA06g039410.3C	-5.28213897	Down
BraA05g016300.3C	-5.182177674	Down	BraA06g044700.3C	-5.28213897	Down
BraA09g018680.3C	-5.182177674	Down	BraA08g000170.3C	-5.28213897	Down
BraA08g022310.3C	-5.129710254	Down	BraA08g026820.3C	-5.28213897	Down
BraA10g022010.3C	-5.129710254	Down	BraA03g025720.3C	-5.225555442	Down
BraA09g016490.3C	-5.123760988	Down	BraA06g029760.3C	-5.225555442	Down
BraA02g033150.3C	-5.07526247	Down	BraA07g015800.3C	-5.225555442	Down
BraA09g061510.3C	-5.056646792	Down	BraA09g054780.3C	-5.225555442	Down
BraA04g003000.3C	-5.018678942	Down	BraA09g002820.3C	-5.190508495	Down
BraA06g014350.3C	-5.018678942	Down	BraA08g021310.3C	-5.18656131	Down
BraA01g017450.3C	-4.959785252	Down	BraA02g045000.3C	-5.166661753	Down
BraA03g042980.3C	-4.959785252	Down	BraA09g016490.3C	-5.13799241	Down
BraA05g035590.3C	-4.959785252	Down	BraA06g036590.3C	-5.105261208	Down
BraA08g020170.3C	-4.929411603	Down	BraA07g031060.3C	-5.105261208	Down
BraA01g006200.3C	-4.910098128	Down	BraA07g026050.3C	-5.059746549	Down
BraA06g010150.3C	-4.908152484	Down	BraA06g010140.3C	-5.044983196	Down
BraA05g011600.3C	-4.898384708	Down	BraA03g010890.3C	-5.041130871	Down
BraA05g013040.3C	-4.898384708	Down	BraA05g006700.3C	-5.041130871	Down
BraA03g030540.3C	-4.882617392	Down	BraA06g033290.3C	-5.041130871	Down
BraA07g026520.3C	-4.850556183	Down	BraA06g020020.3C	-5.007964007	Down
BraA02g017840.3C	-4.841522371	Down	BraA09g001210.3C	-5.007964007	Down
BraA06g031270.3C	-4.83425437	Down	BraA07g027360.3C	-4.977828289	Down
BraA06g035520.3C	-4.83425437	Down	BraA03g021530.3C	-4.944269331	Down
BraA08g021340.3C	-4.83425437	Down	BraA02g027120.3C	-4.903627347	Down
BraA01g009400.3C	-4.767140175	Down	BraA09g052950.3C	-4.903627347	Down
BraA07g038210.3C	-4.767140175	Down	BraA10g011210.3C	-4.903627347	Down
BraA08g020180.3C	-4.767140175	Down	BraA06g010150.3C	-4.867101471	Down
BraA09g025340.3C	-4.767140175	Down	BraA08g004500.3C	-4.867101471	Down
BraA06g010140.3C	-4.755644536	Down	BraA04g021340.3C	-4.848485793	Down
BraA02g040390.3C	-4.74737692	Down	BraA08g017170.3C	-4.829626766	Down
BraA08g030080.3C	-4.741504248	Down	BraA10g019300.3C	-4.669162093	Down
BraA04g021750.3C	-4.739395184	Down	BraA02g022450.3C	-4.645829589	Down
BraA09g041650.3C	-4.725319999	Down	BraA07g009310.3C	-4.633787763	Down
BraA06g002680.3C	-4.722960726	Down	BraA07g037640.3C	-4.614935582	Down
BraA09g002820.3C	-4.720597589	Down	BraA09g038900.3C	-4.558979176	Down
BraA09g001320.3C	-4.696750847	Down	BraA03g042730.3C	-4.535895562	Down
BraA07g027360.3C	-4.692949276	Down	BraA05g009060.3C	-4.488589848	Down
BraA03g023610.3C	-4.664329369	Down	BraA08g023200.3C	-4.488589848	Down
BraA01g037380.3C	-4.660224971	Down	BraA10g020710.3C	-4.488589848	Down
BraA05g009820.3C	-4.658854239	Down	BraA08g003730.3C	-4.464342301	Down
BraA06g010180.3C	-4.641255734	Down	BraA07g036660.3C	-4.445197252	Down
BraA03g017510.3C	-4.622750265	Down	BraA07g035640.3C	-4.389054174	Down
BraA06g010160.3C	-4.607483509	Down	BraA03g012750.3C	-4.363058966	Down
BraA03g001030.3C	-4.571219965	Down	BraA01g032370.3C	-4.354288756	Down
BraA03g060650.3C	-4.544747753	Down	BraA02g006080.3C	-4.318664846	Down
BraA03g006750.3C	-4.540025343	Down	BraA06g002680.3C	-4.28213897	Down
BraA08g027490.3C	-4.52166414	Down	BraA06g013720.3C	-4.28213897	Down
BraA06g029890.3C	-4.514374104	Down	BraA07g038440.3C	-4.28213897	Down
BraA03g041150.3C	-4.462285593	Down	BraA08g017600.3C	-4.28213897	Down
BraA07g033480.3C	-4.404570095	Down	BraA07g039320.3C	-4.263523292	Down
BraA09g011350.3C	-4.377983093	Down	BraA04g003800.3C	-4.24347735	Down
BraA02g006310.3C	-4.344034237	Down	BraA02g007790.3C	-4.225555442	Down

BraA08g020160.3C	-4.338296876	Down	BraA05g002570.3C	-4.225555442	Down
BraA09g003790.3C	-4.335652154	Down	BraA06g010190.3C	-4.219606176	Down
BraA03g010480.3C	-4.329019062	Down	BraA01g006200.3C	-4.210084033	Down
BraA01g038780.3C	-4.313422207	Down	BraA08g018490.3C	-4.19967681	Down
BraA02g019240.3C	-4.308231177	Down	BraA08g030400.3C	-4.196409096	Down
BraA02g039660.3C	-4.289092878	Down	BraA09g009510.3C	-4.196409096	Down
BraA05g040300.3C	-4.281713347	Down	BraA02g003350.3C	-4.178634394	Down
BraA03g012220.3C	-4.24929187	Down	BraA06g010180.3C	-4.149787934	Down
BraA08g021310.3C	-4.242719216	Down	BraA08g018620.3C	-4.143884282	Down
